# Infant growth disparity in the Khanh Hoa province in Vietnam: a follow-up study

**DOI:** 10.1186/1471-2431-10-62

**Published:** 2010-08-23

**Authors:** Arild Vaktskjold, Đoàn Văn Trí, Dương Trọng Phỉ, Torkjel Sandanger

**Affiliations:** 1 Nordic School of Public Health, Sweden [a subsidiary of the Nordic Council of Ministers, Denmark; 2 Pasteur Institute, Nha Trang, Vietnam; 3 Norsk Institutt for Luftforskning, Tromsø, Norway

## Abstract

**Background:**

Surveys in Vietnam have indicated that wasting and stunting have been prevalent among children, but the country is undergoing rapid socio-economic changes and little has been known about the relative situation in the different areas of the country. In 2006, the WHO introduced new growth standards applicable to all infant and child populations, which facilitates for improved assessments of the prevalence of growth impairment, independent of time, place and ethnicity. The aim of our study was to assess the growth of singleton infants delivered at term in three main birth clinics in the Khanh Hoa province in Vietnam by using the new WHO standards as reference, and the association between growth and some maternal, birth and health factors.

**Methods:**

A cohort of 237 singleton infants born in the period May-July 2005 in three main delivery clinics in the Khanh Hoa province were observed prospectively. Their anthropometrical measures a year later were compared to the WHO sex-specific growth standards for weight-for-age, length-for-age, weight-for-length, and BMI-for-age. These measures were analysed as dependent outcomes using multiple linear regression models including the following independent factors: urban vs. rural birth, 1-minute Apgar score, weight and length at birth, duration of lactation, ever had diarrhoea, dengue fever, pneumonia or dysentery, and maternal age, height, gestational duration and parity.

**Results:**

Compared to the standard distributions, 79% were below the median for weight-for-length; 18.0% were within the 5^th ^percentile for length-for-age, 9.6% for weight-for-age, 20.3% for weight-for-length, and 19.8% for BMI. A lower length- and weight-for-age were statistically associated with being born rurally.

**Conclusions:**

In this delivery-clinic based sample of children in the Khanh Hoa province in Vietnam, the proportions within the WHO-standard 5^th ^percentiles for length-for-age, weight-for-length and BMI in late infancy were 3-4 times higher than expected, which indicate that deficient growth is prevalent. The infants born in a rural area had a lower weight- and length-for-age than their urban counterparts, independent of diarrhoea.

## Background

Human growth is sensitive to the environment, and a child may be inhibited from reaching its genetic growth potential if nutritional, socio-economic, health, or environmental factors are unfavourable prenatally or postnatally [1,2]. In general, the physical growth rate of a child is rapid the first eight months and thereafter decreasing with age until the adolescence growth spurt [3-5]. Impaired stature growth in early life is associated with poor functional outcomes in later life and these children do usually not catch up in growth [6,7].

The World Health Organisation (WHO) Working Group on Infant Growth views anthropometric assessments of infants useful to assess nutritional adequacy and the impact of illness [8]. Based on child growth in multiple study centres, the WHO developed growth standards to facilitate for determination whether *physiological needs for growth and development are met during important childhood periods *[[5], page 1], which were made public in 2006. The standards intend to show how healthy, breastfed children living under favourable conditions should grow in all populations, regardless of time and place. Thus, deviations from the standard pattern can be considered evidence of abnormal growth [5], and are used to diagnose wasting, stunting, over- and underweight [9].

There are several measures of infant and child growth and size. Each measure has its weaknesses and strengths, and no measure is ideal during a long period of growth as the correlation between height and weight varies [10]. Thus, the ideal measure for prediction and comparison of growth should be highly correlated with weight and uncorrelated with height [11]. Since height (length) is uncorrelated with weight, it provides a good measure of stunted growth for age, such as in long term protein malnutrition or dietary inadequacy. Low weight-for-length has been used as a measure of wasting, which can result from weight loss or failure to gain weight. The WHO growth standards include weight, height (length) and body mass index (BMI) for age; and weight-for-length. When more than five percent in a population fall below the 5^th ^percentile of the standard, inferior growth is present [5].

According to estimates by the WHO (2006) about 1/3 of children under age five in Vietnam are stunted and/or underweight for age [12]. Anaemia, malnutrition and hookworm infestation have been widespread, especially in the rural areas of the country [13-15], and the high environmental contamination and pesticide use is reflected in the breast milk [16]. In 2006, the government announced an anti-malnutrition programme to reach the national goal of less than 25 percent stunted growth and less than 20 percent underweight in its child population by 2010 [17]. A plan to reduce child malnutrition had also been launched in 2001 [14]. Thus, studies using the new WHO standards are warranted to assess the situation and developments concerning the growth of children in Vietnam, also provincially.

The aim of this study was to assess whether the growth of singleton infants born at term in three main delivery hospitals in the Khanh Hoa province in Vietnam fell in line with the percentile distributions in the new WHO standards; the prevalence of impaired growth; and the association between different maternal and infant factors and the infants' growth. The specific study questions were: 1) was the proportion within the standard 5^th ^percentile for weight-for-length and weight, height (length) and BMI-for-age, respectively, different than 5 percent; and 2) were any of the following factors important determinants of the size of the children in late infancy: gestational duration, rural or urban district of birth, 1-minute Apgar score, weight and length at birth, duration of lactation, anaemia at follow-up, diarrhoea, pneumonia, dysentery or dengue fever in infancy (grouped as one factor), and maternal age, height and parity.

## Methods

### Enrolment and Data Collection

The study was carried out in the province capital Nha Trang located at the south-central coast (363 000 inhabitants) and the nearby inland rural district Dien Khanh (126 000). There were 32 delivery wards in Nha Trang and 26 in Dien Khanh. In 2005, the number of births was 5972 and 1925, respectively.

In the three main delivery units (two in Nha Trang [the obstetric department at the provincial hospital and the public delivery clinic] and one in Dien Khanh [the obstetric clinic at the district hospital]), a total of 245 consecutive women in routine third-trimester pregnancy care were invited to participate in a follow-up study of their newborns; five (2%) chose to not participate. The enrolled women delivered 242 newborns in the period May-July 2005, whereof 237 were live born and singleton (128 in Nha Trang and in 109 in Dien Khanh). The following baseline data were collected about each birth in a standard form: the name of the clinic, maternal age, weight, height, parity, gestational age (determined by date of last menstruation), pregnancy and delivery complications and delivery date, and neonatal 1-minute Apgar score, sex, birth weight, length, head circumference and presence of congenital malformations. Additional details about the enrolment and baseline data collection have previously been published [18].

After a year (in May-July, 2006) the weight and length of each infant was measured and the mother asked about diseases and lactation while visiting the local health centre. A home visit was carried out to those who did not appear at the centre. The data were supplemented by clinical observations of diarrhoea and diseases in infancy. The weight of the child was measured to the nearest hectogram using Health Scale model TZ 120 and the length to the nearest centimetre in recumbent position. The latter was measured using the tool made and used by the National Programme of Nutrition for Children in Vietnam. The data collection was carried out by nurses and supervised by the Pasteur Institute in Nha Trang; initiated in co-operation with the University in Tromsø and Norsk Institutt for Luftforskning, Norway.

### Birth information of the study sample

The mean birth weight at baseline (51% boys) was 3201 grams (95% confidence interval [CI]: ± 46 grams) and length 48.4 cm (CI: ± 0.3 cm). Four (1.7%) of the infants weighed less than 2500 grams, but all had at least 37 weeks of gestation, and 3 were born with an anomaly. The mean maternal age was 27.8 (CI: ± 0.7) years (mode = 27). Some 44 percent of the enrolled women delivered for the first time. The main birth characteristics are presented in Table 1.

**Table 1:** Characteristics of the infants and mothers at baseline (n = 237)

	Mean	**SD**^**1**^	Range	Missing data
Birth weight (grams)	3202	360	2000-4000	4 (1.7%)
Birth length (cm)	48.4	2.5	40-55	3 (1.3%)
Gestational length (weeks)	39.6	0.8	37-42	4 (1.7%)
1-minute Apgar score	9.9	0.4	8-10	2 (0.8%)
Maternal age (years)	27.8	5.3	18-49	0 (0.0%)
Number previous deliveries	0.65	0.67	0-3	0 (0.0%)
Maternal height (cm)	155	4.3	144-167	3 (1.3%)

^1 ^Standard deviation

### Analyses

The infant size at the age of follow up was analysed using weight- and length-for-age, weight-for-length, and BMI (weight/length^2^)-for-age as measures. The measures of each infant were compared with the WHO sex-specific growth standards for age in months and weight-for-length to determine where they fell in the standard percentile distribution [5], and the proportion within the 5^th ^percentile estimated. In addition, a linear multiple regression model was set up using each of the four measures as outcomes to analyse their association with the following *a priori *chosen factors: gestational duration, district of delivery, 1-minute Apgar score, weight and length at birth, duration of lactation, anaemia at follow-up, birth defects, diarrhoea, pneumonia, dysentery or dengue fever (grouped as one factor) before and after age 6 months, respectively, and maternal age, height and parity. The parameter estimates were adjusted for infant age at follow-up (in days) and sex. The significance level was set at 5 percent in each test and 95 percent confidence intervals (CI) presented. The goodness-of-fit of the models was visually evaluated by comparing normal probability plots and residual plots for each included variable.

### Ethical considerations

The study was approved by the Ministry of Health in Viet Nam and the University of Tromsø in Norway, while the Ethical Council of the Ministry of Health in Viet Nam approved the method of data collection. Each participating woman was presented an informed consent form before delivery and at follow-up, which were voluntary to sign.

## Results

Some 221 (93.2%) of the newborns were located at follow up. Their mean age was 369 days (range 320-413). The unadjusted length varied from 65 to 96 cm and weight from 5.8 to 14.0 kg. On average, the length had increased by 26.8 cm (range 15-53) and the weight by 5.9 kg (CI: 3.3-10.1). Boys had a higher weight, length, weight-for-length and BMI than girls. Twelve (5.4%) of the infants had been clinically observed with diarrhoea and one with dysentery, pneumonia and dengue fever, respectively. Of these, 5 were diagnosed before age 6 months. 29 women (15%) had terminated lactation before the follow-up. The mean duration and range of breastfeeding, outcome measures and proportion of data missing at follow-up are presented in Table 2. A blood sample was collected from 189 infants at follow-up; of these 21 (11.1%) had anaemia. The perinatal characteristics (listed in Table 1) of the infants lost to follow-up did not differ statistically from those studied.

**Table 2:** Mean age, duration of breast-feeding and anthropometric measures of infants at follow-up (n = 221)

	Mean	**SD**^**1**^	Range	Missing data
Age (days)	369	16.8	320-413	0 (0.0%)
Duration of lactation (months)	11.3	2.2	0.5-12	27 (12.2%)
Length (cm)	75.2	5.2	65-96	1 (0.5%)
Weight (kg)	9.1	1.2	5.8-14.0	0 (0.0%)
Weight-for-length (kg/m)	12.2	1.4	8.9-17.7	1 (0.5%)
Body mass index (kg/m^2^)	16.3	2.2	9.4-22.4	1 (0.5%)

^1 ^Standard deviation

A higher proportion of the infants was in the low end for weight, and in both the low and high ends for length compared to the standard distributions. 18.0 (95% CI: 13.0, 23.0) percent were within the standard 5^th ^percentile for length-for-age, 9.6 (5.7, 13.8) percent for weight-for-age, 19.8 (14.7, 25.1) percent for BMI, and 20.3 (14.9, 25.7) percent for weight-for-length. Compared to the standard 25^th ^percentile, 36.7 percent were below for weight, 39.2 percent for length, and 38.2 percent for weight-for-length. The median infant had a length-for-age similar to the standard but a lower weight-for-age and weight-for-length. The proportion above the 95^th ^percentile was similar to the standard for weight-for age, but higher for length-for-age. The detailed distributions of growth for age compared to the standards for all four outcome-measures are presented in Table 3.

**Table 3:** The cumulative distribution of the infants at follow-up based on the WHO standard growth percentiles for weight-for-age, length-for-age, weight-for-length, and body mass index-for-age [5].

Percentile in WHO-standard	Weight-for-age		Length-for-age		Weight-for-length		Body mass index	
	**N**	**%**	**N**	**%**	**n**	**%**	**n**	**%**
≤5^th^	22	9.6	39	18.0	44	20.3	43	19.8
≤25^th^	80	36.7	85	39.2	83	38.2	78	35.9
≤50^th^	138	63.3	119	54.8	128	79.3	116	53.9
≤95^th^	209	95.9	178	82.0	199	91.7	196	90.3
Total	218	100	217	100	217	100	217	100

Duration of breastfeeding and anaemia were not associated with the outcome in any of the four models and were removed to decrease the number of observations lost in the analyses due to missing values. The adjustment factors and remaining study factors explained 17 percent of the variance in length, 25 percent of weight, 14 percent of weight-for-length, and 6 percent of BMI. Infants born in Dien Khanh (rural) were 2.2 cm shorter (CI: 0.5-4.0 cm) and weighed 550 grams less (142-957 grams) for age than those born in the city, adjusted for the other factors. The adjusted weight increased on average with 43 grams (6-80 grams) per cm increase in maternal height in the range 144-167 cm. No study factors were statistically associated with weight-for-length and BMI. Birth defects were not included in the multiple analyses as only two of the children available at follow-up were inflicted (cleft lip and palate, and excess number of fingers). The parameter estimates (beta) for the study factors for each of the four study outcomes are presented in Table 4.

**Table 4:** The adjusted linear parameter estimates (beta) and their 95% confidence limits of the studied factors, regressed on length-for-age, weight-for-age, weight-for-length, and body mass index (BMI)-for-age at follow-up ^a^

	**Length-for-age**^**b**^	**Weight-for-age**^**c**^	**Weight-for-length**^**d**^	BMI-for-age
Intercept	30.2 (-7.9, 68.2)	-1.1 (-10.0, 7.9)	5.9 (-5.3, 17.1)	17.4 (0.01, 34.9)
District of birth ^e^	-2.2 (-4.0, -0.5)	-0.5 (-1.0, -0.1)	-0.35 (-0.86, 0.16)	0.03 (-0.76, 0.83)
Apgar score	0.8 (-1.0, 2.6)	0.1 (-0.3, 0.5)	0.01 (-0.52, 0.53)	-0.13 (-0.95, 0.69)
Maternal age	-0.1 (-0.4, 0.1)	-0.0 (-0.1, 0.0)	-0.01 (-0.05, 0.04)	0.01 (-0.06, 0.08)
Parity	-0.2 (-1.4, 1.1)	-0.0 (-0.3, 0.3)	0.04 (-0.34, 0.41)	0.11 (-0.47, 0.70)
Maternal height (cm)	0.1 (-0.0, 0.3)	0.0 (0.0, 0.1) ^f^	0.03 (-0.01, 0.08)	0.01 (-0.06, 0.09)
Birth weight (g)	0.9 (-1.3, 3.0)	0.3 (-0.2, 0.8)	0.29 (-0.33, 0.91)	0.12 (-0.86, 1.10)
Birth length (cm)	-0.0 (-0.3, 0.3)	-0.0 (-0.1, 0.1)	-0.02 (-0.11, 0.08)	-0.02 (-0.17, 0.13)
Diarrhoea first 6 months ^g^	2.4 (-1.9, 6.7)	-0.5 (-1.5, 0.5)	-1.00 (-2.22, 0.30)	-1.70 (-3.67, 0.27)
Diarrhoea after age 6 months ^g^	-2.7 (-5.7, 0.4)	-0.6 (-1.3, 0.2)	-0.37 (-1.27, 0.54)	0.00 (-1.41, 1.41)
*R-square *^*a*^	0.174	0.246	0.141	0.063

^a ^All four models were adjusted for sex. ^b ^Length in cm. ^c ^Weight in kg. ^d ^Weight in kg and length in metres, adjusted for age at follow-up. ^e ^Nha Trang = 0 and Dien Khanh (rural) = 1 in the models. ^f ^In grams: 43 (6-80). ^g ^Includes diagnoses of diarrhoea, pneumonia, dysentery or dengue fever.

The tolerance was at least 0.5 for every factor in each of the final models, which indicates that multi-colinearity was not present. Ten (4.5%) of the observations at follow up had missing values for one or more of the covariates. Running the models post hoc without the two most outlying observations of weight and length at follow up did not change the parameter estimates, but strengthened the association between district of birth and weight.

## Discussion

A higher proportion of the cohort was in the low end of the standard distribution than expected for all four outcome-measures. On an individual basis, a low weight and/or length for age is either due to normal variation in growth or a deficit in growth. The employed WHO growth standards were based on the growth of healthy infants and children in several countries, including developing countries [5], and include the normal variation. Thus, our findings indicate that stunted growth was prevalent, and that also wasting was present. Studies in other countries have shown that children short for their age in early life are at elevated risk of poor functional outcomes later in life and that they tend to not catch up in growth [6,7]. Low weight-for-age is an important risk factor for death due to diarrhoea, malaria, measles and pneumonia [19]. Using the new WHO standards probably highlights the proportion of infants at elevated risk better than the previous standards [9].

Nation-wide surveys of the prevalence of malnutrition in the age group 0-5 years found a substantial reduction in Vietnam between 1990 and 2004. Nevertheless, the prevalence of stunting was estimated to 31 percent and wasting 8 percent in 2004 [14]. A survey of the age group 6 to 17.9 months using the new WHO growth standards found that 19 percent were stunted and 14 percent wasted [9]. Considering that stunting tends to reflect long term malnutrition, and that the proportion of children in Asia and Vietnam falling below the mean standard growth therefore has tended to increase with age during the first few years of life [9,20], the prevalence of stunting in infancy in our Khanh Hoa cohort appears to be relatively high also in a Vietnam perspective. However, our study is one of the first contributions to the knowledge base concerning Vietnam after the new WHO standards were introduced.

To examine the infants in the low end of the standards closer, we carried out post-hoc analyses (by multiple logistic regression) of the risk of being within the 5^th ^percentile of the growth standards for the study factors listed in Table 4. The results of the analyses suggested that diarrhoea during the first six months increased the risk of low BMI-for-age and weight-for-length, and that diarrhoea after 6 months of age increased the risk of low weight-for-age. Weight-for-age was also associated with birth weight, while a low length-for-age was associated with being born rurally. Thus, diarrhoea apparently played a larger role for infants to fall below the 5^th ^percentile of the standards than on the overall growth in the cohort, but stunting appears to be more common in the rural area independent of diarrhoea.

More studies are needed to map the situation, and to evaluate whether the Vietnam government's anti-malnutrition programme has had an effect and their target for the under-five population been reached [17]. Our finding of a lower height- and weight-for-age among the rurally born infants is important in that perspective; a finding that fell in line with findings in the past concerning the difference in rural and urban areas [13,14]. A poorer health in rural than urban populations has an impact on many people in Vietnam considering that 70 percent of the population of more than 80 million live in rural areas, including approximately one million infants.

Studies in Vietnam have reported that the growth of Vietnamese infants began to lag behind a Western reference population from age four-five months [4,21]. The authors suggested that the introduction of inadequate complementary foods from that age was the explanation. A study of antenatal feeding by Nga and Weissner (1986) in the northeast of the country concluded that inadequate feeding and poor feeding during diarrhoea is an explanation of an observed drop in mean growth between 6 and 12 months of age [22]. This is also a reasonable explanation for our findings in Khanh Hoa.

Nevertheless, the overall mean weight and length at follow-up were higher than that reported from a study in Hanoi a decade ago [4], and the same as in a recent sample of infants in a rural setting with normal birth weight [21]. Compared to the findings by Fort (1973) in a sample of healthy Vietnamese children 40 years ago [3], the average infant in our study was heavier but shorter. Pooling an urban and a rural population to assess determinants, as we did, is justifiable as a vast proportion of the variability in growth is due to individual differences [5].

We found no association between length at birth and weight and length at follow-up, which is in accordance with findings that infants small at birth tend to catch up during the first year of life [23]. The difference in length and weight between boys and girls was comparable to that revealed in Vietnam earlier [4]. The apparent explanation of the positive association between the infants' weight and maternal height was the slight positive correlation between the mothers' and infants' height. Bryan and Hindmarsh (2006) reported that the unadjusted positive correlation between maternal height and infant length increases with the children's age. The correlation coefficient in their study was 0.30 at age 12 months, compared to 0.18 at follow-up in our study sample [24]. An association between parity (number of children in the household) and stunting, as reported by Khan et al. (2007), did not appear in our cohort [14]. However, when evaluating the precision of the parameter estimates one needs to consider that most of the variance in length and weight was not explained by the factors included in the multiple analyses.

Sixteen newborns (6.8%) were lost to follow up because the mothers did not live at the address given to the birth clinic. It is common that pregnant women in Vietnam travel to their home district and their mothers, and deliver there. Our study sample met well the baseline inclusion criteria behind the WHO growth standard [5]. Namely, only singleton births delivered at term were included in the study sample; smoking in pregnancy was more or less absent [18]; and our data show that breastfeeding was common. Some 85 percent were breastfeeding at the time of follow-up, and only 4.6 percent breastfed less than 6 months. We did not collect data on the extent of *exclusive *breastfeeding, but it is known that exclusive breastfeeding beyond 6 months is negatively associated with infant growth in both weight and length [5,8,23].

Measurements of length and weight are often difficult to obtain in a primary care setting [8], but no information bias was apparent. As seasonal variation in growth is common in developing countries [25], it was a strength that the studied infants were born within a few months of each other. In addition, the age around one is critical in terms of child growth and the age when growth retardation appears. By using the actual age at the time when the follow-up anthropometric measurements were carried out, the data facilitated well for assessment of growth based on the WHO standards. On the other hand, the spread in infant age at follow-up and the small sample size did not provide for estimating the variance or z-score at each age-point in months. The z-score has also been commonly used to assess wasting and stunting [4,14], where wasting has been defined as less than two standard deviations below the median of the reference standard [20]. Due to this shortcoming, we chose to present ranges instead of confidence intervals around the means (Tables 1 and 2). The main limitation of our study was the relatively small sample size, which is reflected in the confidence intervals of the estimates. Thus, the estimates of the exact magnitude of stunting, wasting, and of the difference between rural and urban born infants in the studied cohort have low precision. On the other hand, despite the small sample size the study clearly revealed that stunting was prevalent and that the length-for-age was lower for children born in the rural district.

As the study sample was recruited from the three main delivery clinics, it represented well infants delivered in the main clinics in the province, but was thereby less representative of children born in the numerous small delivery wards. In addition, the rural population was overrepresented in the sample compared with the proportion in the newborn population in the province as a whole. Thus, in terms of the magnitude of the estimates, the external validity of the study was limited. On the other hand, that a relatively large difference in birth weight was detected between rural and urban areas was likely not influenced. Furthermore, there was a relatively high proportion within the low tails of the standard distributions even though only children born in the main delivery clinics were included. The proportions in the provincial population as a whole might be even higher.

## Conclusions

The distributions of length-for-age, weight-for-length and BMI in late infancy of children born at term in central delivery clinics in the Khanh Hoa province in Vietnam deviate from the WHO standards. The proportions within the 5^th ^percentiles of the standards were 3-4 times higher than expected. Our findings indicate that deficient growth is prevalent in this province, and that infants born in a rural area had a lower weight and length in late infancy than their urban counterparts - independent of the occurrence of diarrhoea. Interventions to prevent deficient growth early in life are still needed in Vietnam - also in the Khanh Hoa province.

## Competing interests

Our interpretation of the data or presentation of the findings has not been influenced by our personal or financial relationship with other people or organisations.

## Authors' contributions

AV analysed and interpreted the data, and wrote the manuscript. DVT lead the enrolment of participants, data collection and data entering. DTP organised the investigation and supervised the data collection. TS designed and supervised the study and data collection, and helped drafting the manuscript. All authors have read and approved the final manuscript.

## Pre-publication history

The pre-publication history for this paper can be accessed here:

http://www.biomedcentral.com/1471-2431/10/62/prepub
